# Plastid genome of stonecrop *Hylotelephium verticillatum* (Sedoideae; Crassulaceae): insight into structure and phylogenetic position

**DOI:** 10.1080/23802359.2020.1788464

**Published:** 2020-07-09

**Authors:** Seon-Hee Kim, Seung-Chul Kim

**Affiliations:** Department of Biological Sciences, Sungkyunkwan University, Suwon, Gyeonggi-do, Republic of Korea

**Keywords:** Chloroplast genome, Sedoideae, *Hylotelephium verticillatum*, *Sedum* sensu lato, Crassulaceae

## Abstract

The first complete chloroplast genome sequence of *Hylotelephium verticillatum*, was reported in this study. The plastome size was 151,398 bp in total length, with one large single copy (LSC; 82,951 bp), one small single copy (SSC; 16,839 bp), and two inverted repeat (IR) regions (IRa and IRb, each with 25,804 bp). The overall GC content was 37.8% and the genome contained 128 genes, including 85 protein-coding, 37 transfer RNA, and 6 ribosomal RNA genes. Phylogenetic analysis of 15 representative plastomes within the family Crassulaceae suggests that *H. verticillatum* is sister to congeneric *H. ewersii*.

*Hylotelephium* H.Ohba is a genus of flowering plants in the stonecrop family Crassulaceae and includes approximately 30 species in Northern Hemisphere (Mayuzumi and Ohba 2004). With a few additional genera in the subfamily Sedoideae, it was previously recognized under highly polyphyletic a catchall genus *Sedum* (Praeger 1921; ‘t Hart 1982). The infrafamilial classification of Crassulaceae based on recent morphological and molecular phylogenetic studies provided several new insights into delimitation of subfamilies and genera (‘t Hart 1995; Mayuzumi and Ohba 2004; Gontcharova et al. 2006). Specifically, *Hylotelephium* was confirmed as one of segregate genera from *Sedum s.l.* (i.e., *Rhodiola*, *Phedimus*, and *Umbilicus*) and close relationship between *Hylotelephium* and *Orostachys* was suggested (Mayuzumi and Ohba 2004; Gontcharova et al. 2006). It was also shown that *Hylotelephium* is not monophyletic and is closely related to polyphyletic *Orostachys* and two other genera, *Meterostachys* and *Sinocrassula*, in the clade of ‘Hylotelephium’. Several *Hylotelephium* diagnostic features include stipitate or attenuate ovaries, flat broad leaves, compound corymbose inflorescences, and non-yellow petals (Ohba 1977). In Korea, six taxa of *Hylotelephium* occur and *H. verticillatum* (L.) H.Ohba, a herbaceous perennial, is commonly distributed throughout the Korean peninsula (Lee 2006). Little is known for plastid genome structure and organization among congeneric species of *Hylotelephium*. Thus, we sequenced the complete plastome of *H. verticillatum* and assessed its phylogenetic position within the East Asian Sedoideae.

The total genomic DNA (Voucher specimen: SKK-HV180705006; 38°11′05.60″N 128°05′44.3″E) was isolated using the DNeasy plant Mini Kit (Qiagen, Carlsbad, CA) and sequenced by the Illumina HiSeq 4000 platform (Illumina, San Diego, CA, USA) at Macrogen Corporation (Seoul, Korea). A total of 35,607,680 paired-end reads were obtained and assembled *de novo* with Velvet v. 1.2.10 using multiple *k*-mers (Zerbino and Birney 2008). The plastome of *Nicotiana tabacum* (NC_001879) was used as the reference for initial gene annotation with Geneious R11.1.5 (Biomatters Ltd., Auckland, New Zealand). Then, gene annotation was manually corrected for start and stop codons and for intron/exon boundaries. The transfer RNAs (tRNA) were predicted using ARAGORN v 1.2.36 (Laslett and Canback 2004) and RNAs (rRNA) were identified using RNAmmer 1.2 Server (Lagesen et al. 2007).

The complete plastome sequence of *H. verticillatum* (GenBank: MT558730) was 151,398 bp in total length and is composed of large single copy (LSC; 82,951 bp), small single copy (SSC; 16,839 bp), and two inverted repeats (IRa and IRb, each with 25,804 bp). The overall GC content was 37.8%, and those of LSC, SSC and IR regions were 35.8%, 31.6%, and 43%, respectively. The chloroplast genome included 128 genes, including 85 protein coding genes, 37 transfer RNA, and six ribosomal RNA genes. The *ycf*1 gene in the junction region of IRb and SSC was annotated as a pseudogene , which was formed by incomplete duplication of the normal copy of *ycf*1 in the IRa and SSC junction region. The *ycf*15 gene was also observed as a pseudogene due to the generation of an internal stop codon. A total of 16 genes were duplicated in the inverted repeat regions, including seven tRNA genes (*trn*I-CAU, *trn*L-CAA, *trn*V-GAC, *trn*L-GAU, *trn*A-UGC, *trn*R-ACG, and *trn*N-GUU), three rRNA genes (rRNA5, rRNA23, and rRNA16), and six protein-coding genes (*rpl*2, *rpl*23, *ycf*2, *ndh*B, *rps*7, and *rps*12). Fourteen genes (*atp*F, *ndh*A, *ndh*B, *pet*B, *pet*D, *rpl*16, *rpl*2, *rpo*C1, *rps*16, *trn*A-UGC, *trn*G-GCC, *trn*K-UUU, *trn*L-GAU, *trn*L-UAA, and *trn*V-UAC) contained one intron, while *clp*P, *rps*12, and *ycf*3 each contained two introns. Exceptional genomic structural change, such as inversions, transpositions, and gene duplications and losses, in the *H. verticillatum* plastome was not found compared to closely related genomes.

To assess the phylogenetic position of *Hylotelephium verticillatum,* 15 representative species of Crassulaceae (Gontcharova and Gontcharov 2009), were aligned using MAFFT (Katoh and Standley 2013) and maximum likelihood (ML) analysis with the best-fit model of GTR + F + R2 and 1,000 ultrafast bootstrap replications was performed based on the whole plastome sequences using IQ-TREE v.1.6.7 (Nguyen et al. 2015). *Kalanchoe tomentosa* (subfamily Kalanchoideae) was chosen as an outgroup. In the ML tree (Figure 1), two clades were recognized: one includes the tribes of Sedeae and Sempreviveae and the other includes the tribes of Umbilicieae and Telephieae. The monophyly of Thiede and Eggli’s (2007) Umbilicieae tribe was not supported in this study because *Umbilicus rupestris* was sister to the clade of Telephieae tribe, which contains the three genera *Sinocrassula*, *Orostachys*, and *Hylotelephium* (100% BS). A sister relationship between *Hylotelephium* and *Orostachys* was strongly supported (100% BS), which was further corroborated by sharing the same basic chromosome number (*x*) of 12 (Uhl and Moran 1972). Within the clade of “Telephium”, the ML tree showed that *H. verticillatum* was most closely related to *H. ewersii* with 100% bootstrap support. The overall phylogenetic relationships among species of *Hylotelephium* and their relationships to *Orostachys* require further rigorous phylogenomic studies.

**Figure 1.**
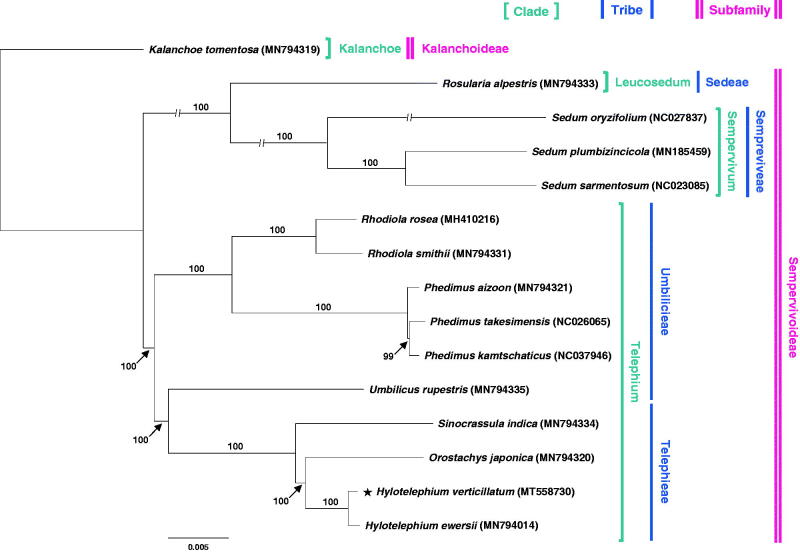
The maximum-likelihood (ML) tree inferred based on complete chloroplast genome sequences from 15 representatives of Crassulaceae. *Kalanchoe tomentosa* (MN794319) was used as an outgroup. The bootstrap support values with 1,000 replicates are shown at each node.

## Data Availability

The data that support the findings of this study are openly available in GenBank, National Center for Biotechnology Information at (https://www.ncbi.nlm.nih.gov/genbank/), with reference number of MT558730.
